# Emerging Antioxidant Paradigm of Mesenchymal Stem Cell-Derived Exosome Therapy

**DOI:** 10.3389/fendo.2021.727272

**Published:** 2021-11-29

**Authors:** Chen Xia, Zhanqiu Dai, Yongming Jin, Pengfei Chen

**Affiliations:** ^1^ Department of Orthopaedic Surgery, Sir Run Run Shaw Hospital, Medical College of Zhejiang University, Hangzhou, China; ^2^ Department of Orthopedic Surgery, Zhejiang Provincial People’s Hospital, People’s Hospital of Hangzhou Medical College, Hangzhou, China; ^3^ Key Laboratory of Musculoskeletal System Degeneration and Regeneration Translational Research of Zhejiang Province, Hangzhou, China; ^4^ Department of Orthopaedic Surgery, The Second Affiliated Hospital, Bengbu Medical College, Bengbu, China; ^5^ Spine Lab, Department of Orthopedic Surgery, The First Affiliated Hospital, Medical College of Zhejiang University, Hangzhou, China

**Keywords:** mesenchymal stem cell, exosome, inflammation, metabolism, oxidative stress

## Abstract

Mesenchymal stem cell-derived exosomes have been under investigation as potential treatments for a diverse range of diseases, and many animal and clinical trials have achieved encouraging results. However, it is well known that the biological activity of the exosomes is key to their therapeutic properties; however, till date, it has not been completely understood. Previous studies have provided different explanations of therapeutic mechanisms of the exosomes, including anti-inflammatory, immunomodulatory, and anti-aging mechanisms. The pathological effects of oxidative stress often include organ damage, inflammation, and disorders of material and energy metabolism. The evidence gathered from research involving animal models indicates that exosomes have antioxidant properties, which can also explain their anti-inflammatory and cytoprotective effects. In this study, we have summarized the antioxidant effects of exosomes in *in vivo* and *in vitro* models, and have evaluated the anti-oxidant mechanisms of exosomes by demonstrating a direct reduction in excessive reactive oxygen species (ROS), promotion of intracellular defence of anti-oxidative stress, immunomodulation by inhibiting excess ROS, and alteration of mitochondrial performance. Exosomes exert their cytoprotective and anti-inflammatory properties by regulating the redox environment and oxidative stress, which explains the therapeutic effects of exosomes in a variety of diseases, mechanisms that can be well preserved among different species.

## Introduction

Mesenchymal stem cells (MSCs) are pluripotent stem cells that can be isolated from various adult or foetal tissues, including fatty tissue, bone marrow, and cord blood (1). Owing to the unique ability to regenerate and differentiate into other cells, MSCs are widely used to treat various diseases (2). They are considered a promising option for the treatment of various types of diseases, such as limb ischemia, skin wound, and cartilage defects (3–5). Despite the potential to replace damaged tissues with a vast array of cells, the potential risks like tumorigenesis and tissue transplant rejection, and ethical issues problems associated with stem cell therapy greatly limit their clinical use (6, 7). This risk persists for a long time (8). In addition, the preparation method of stem cells and the best storage method to maintain the viability of stem cells also pose challenges to their application (9).

The previous studies have shown that the indirect interactions between donor cells and somatic cells rely on the release of exosomes (10). The release of exosomes is precisely regulated by cells. Exosomes are produced in multi-vesicular bodies (MVBs) containing intraluminal vesicles (ILVs) in the cells (11). ILVs are ultimately secreted as exosomes with a size range of 30–200 nm, through the fusion of MVBs to the plasma membrane and exocytosis (11, 12). A series of proteins such as endosomal sorting complex required for transport (ESCRT), transmembrane 4 superfamily (M4SF), Rab protein, and soluble N-ethylmaleimide-sensitive fusion protein attachment protein receptor (SNARE) participate in exosome formation, transportation, transfer, and release (13–15). Almost all cell types can release exosomes *via* the plasma membrane. In addition, exosomes transfer specific protein and genetic information to target cells for intercellular communication (16). The protective lipid bilayer membrane provides exosomes with long-term stability in the microenvironment (17). After release, exosomes can be widely distributed in body fluids (i.e., serum, urine, saliva, breast milk, and semen), and circulate throughout the body through the blood. Target cells can take up exosomes in a variety of ways, including direct fusion, clathrin/caveolin-dependent endocytic pathways, macropinocytosis, phagocytosis, and lipid raft-mediated endocytic pathways (15, 18, 19). The uptake of exosomes by cells is an energy-consuming process (20). In addition, different cells take up exosomes in different ways and often rely on one of the above-mentioned pathways. Therefore, the corresponding uptake behaviour can be inhibited by inhibiting its key proteins such as caveolin and clathrin. At the same time, the pH of the environment is also one of the influencing factors. An acidic environment can enhance the uptake of exosomes, mainly because the acidic environment can increase the expression of caveolin-1 on the surface of exosomes (21, 22). In addition, exosomes may also undergo cell uptake and release cycles in multiple cells to exchange substances. The researchers have also conducted bio-distribution studies on exosomes. After oral administration, exosomes are mainly distributed in the liver, lung, kidney, pancreas, spleen, ovary, colon, and brain, but intravenous administration makes them mainly distributed in the liver, followed by spleen, lung, and gastrointestinal tract (23, 24). Intravenous injection leads to rapid elimination of exosomes from the bloodstream (25), while intranasal administration facilitates the delivery of exosomes to the brain (26, 27). Further in most tissues, macrophages often mediate the uptake of exosomes, and the size of exosomes also affects transportation and bio-distribution. Large-sized exosomes are more likely to accumulate in the bone and liver (28).

The components of exosomes are complex and diverse, including various types of lipids, proteins, mRNAs, and microRNAs, which enable them to act as carriers of various signalling molecules in cells (29–31). Numerous pathological processes have been shown to be related to the exosomes, including tumorigenesis, inflammation, cardiovascular disease, and diabetes (32–35). In addition, exosomes have been reported to perform therapeutic functions and regulate receptor cells through intercellular communication (36, 37). In particular, the application of stem cell-derived exosomes has been proven to maintain functions similar to that of stem cells and avoid the obvious side effects of stem-cell therapy (38). Therefore, exosomes show good therapeutic potential in various diseases. For example, previous reports have confirmed the cell-protective effects of exosomes in the heart, skin, and skeletal muscle diseases (39–41). Recently, the role of exosomes in reducing oxidative and nitrosation damage has attracted a lot of attention. In the pathophysiological process of many diseases, redox environment regulation plays an important role. Numerous studies have evaluated the antioxidant effects of exosome in different disease models, such as the damage caused by hyperglycaemia and obesity (42, 43), alcohol-related brain damage (44), Parkinson’s disease (PD) (45), musculoskeletal diseases (e.g., intervertebral disc degeneration (IVDD), radiation-induced bone loss, osteoarthritis (OA)) (46–48), liver injury (49), ischemia injuries (50), colitis (51), and skin wounds (41) (**Figure 2**). Further, exosomes can directly alleviate oxidative stress in various types of cells such as glial cells (44), neurons (45), cardiomyocytes (52), endothelial cells (53), immune cells (54), hepatocytes (49), and nucleus pulposus cells (46) *in vitro*.

Oxidative stress plays a key role in the pathophysiology of many diseases, by causing cell damage, inflammation, and metabolic disorders. In all living cells, similar components are responsible for mediating excessive oxidative stress and unbalanced reduction. Therefore, exosomes can regulate these molecular components which can be used to treat different diseases. The functions of exosomes are conserved across species.

## Overview of Oxidative Stress

In 1985, Sie et al. first introduced the concept of oxidative stress to the field of redox biology and medicine (55). Oxidative stress is very common under normal physiological conditions, and low levels of oxidative stress may help prevent ageing (56). Mild oxidative stress does not cause any cell damage. It works synergistically with antioxidants to maintain cell homeostasis and plays a role in host defence, gene transcription, and apoptosis (57).

Antioxidants mainly include antioxidant enzymes, such as catalase (CAT), superoxide dismutase (SOD), glutathione peroxidase (GPX), glutathione-S-transferase, and non-enzymatic antioxidant factors, such as melatonin, carotenoid, and some microelements (57). However, when redox homeostasis is disturbed, peroxides and free radicals are produced, which damage proteins, lipids, and DNA. This process is involved in various diseases, such as cancer (58), PD (59), Alzheimer’s disease (60), colitis (51), diabetes (61), liver diseases (62), and the diseases of the musculoskeletal system (63).

The activation of NADPH oxidase (PHOX) is an important pathway to produce reactive oxygen species (ROS), such as H_2_O_2_ (hydrogen peroxide), $O_{2}^{-}$ (superoxide radical), OH^-^ (hydroxyl radical), and NO^·^ (nitric oxide). PHOX is a protein that transfers electrons across biological membranes. Generally, the oxygen is the electron acceptor, the procedure of the electron transfer reaction produces superoxide. Therefore, the production of ROS is the key biological function of PHOX (64).

Four cytosolic subunits (p47*^phox^*, p40*^phox^*, p67*^phox^*, and the small GTPase Rac1/2) and b558, a membrane-bound flavocytochrome form the PHOX. PHOX is activated after the phosphorylation of the cytosolic subunit p47*^phox^*. PHOX plays an important role in redox signalling, which can protect vascular functions and regulate eukaryotic initiator factor 2α-mediated stress signalling (64). Conversely, overexpression of PHOX results in the production of excessive ROS, triggering cell damage and apoptosis (65).

**Figure 1:**
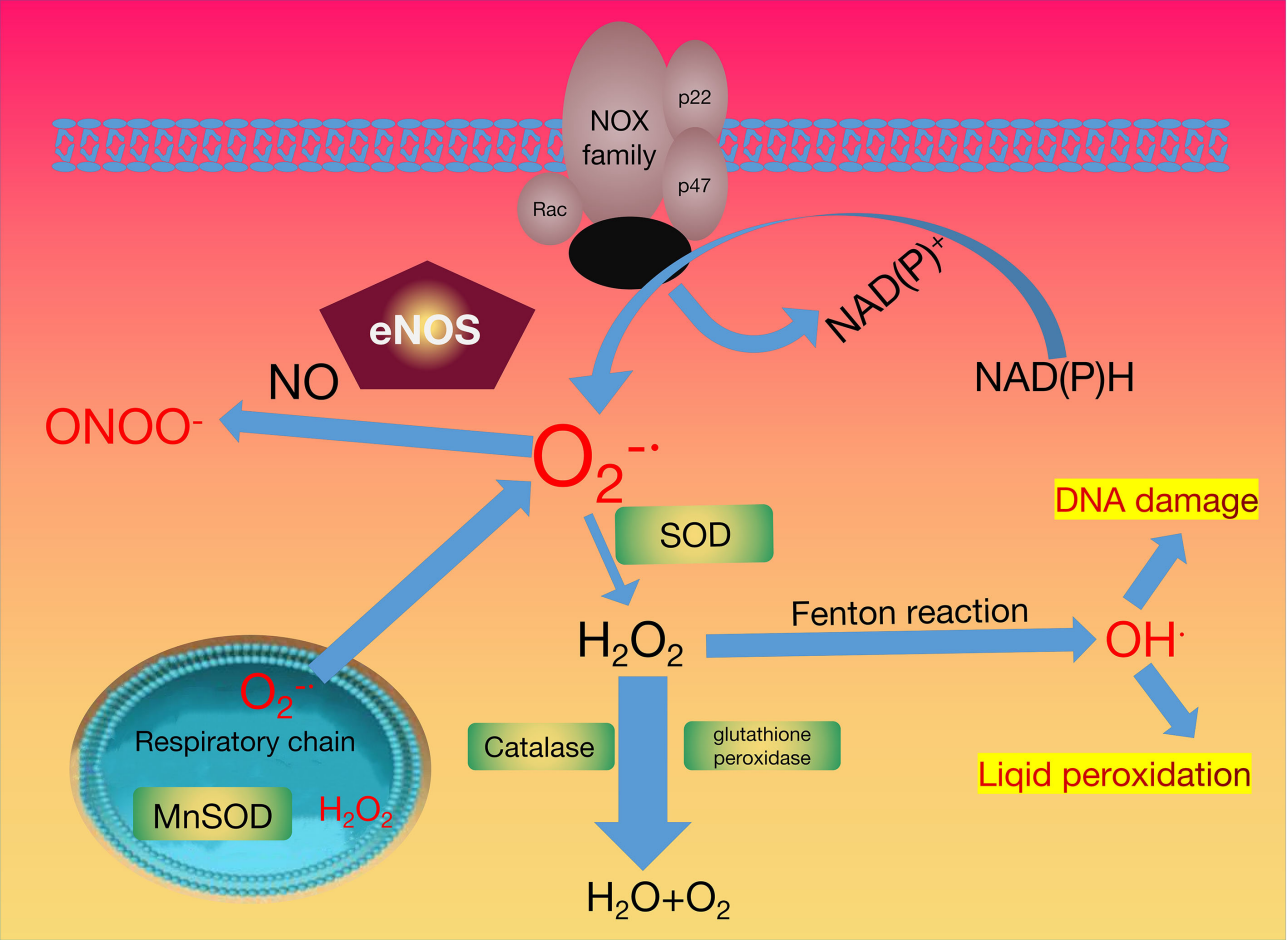
Oxidant and Antioxidant Enzymes System.

The leakage of active oxygen from the mitochondria is considered another source of ROS. Mitochondria are the organelles that produce ATP to provide energy for subcellular processes (66). Several processes involved in cell respiration occur within the mitochondria, including the Krebs cycle, oxidative phosphorylation, and fatty acid β-oxidation. Oxidative phosphorylation plays an important role in the cellular respiration and metabolic pathway and is performed by the inner mitochondrial membrane (IMM) protein complexes and molecules. It mainly includes the following two steps: the electron transport chain (ETC) and the synthesis of ATP. Complex I (NADH-Q reductase, FMN, and Fe-S) is the first proton pump in the electron transport chain. It combines with NADH and transfers the two high-energy electrons on NADH to the FMN prosthetic group to oxidize NADH. FMN is reduced to FMNH2, and then the electrons are transferred to Fe-S. Complex II (succinate-Q reductase, FAD, Cytb) transfers the electrons of FADH2 to the electron transport chain *via* Fe-S, and the relatively high transfer potential energy of the electrons is harnessed. Complex III (Cytochrome reductase, Cytb, Fe-S, Cytc1) catalyses the electrons transfer process from QH2 to cytochrome c. Complex IV (Cytochrome oxidase, CuA, Cyta, Cyta3, CuB) transfers the electrons of the respiratory substrate directly to molecular oxygen through the cytochrome system, and finally generates H_2_O, and the ROS obtained here can be used as a cell signalling molecule or oxidative stress factor. The antioxidant pathways that neutralise ROS are outlined in **Figure 1**.

During the mitochondrial respiratory transport chain, the activation of nicotinamide adenine dinucleotide phosphate oxidase (NOX), xanthine oxidase (XO) generated the superoxide radical anion $\left({\text{O}}_{2}^{-}\right)$ . The superoxide radical anion has two destinations. It can be converted into hydrogen peroxide (H_2_O_2_) by superoxide dismutase (SOD), or interact with nitric oxide (NO^·^) to produce peroxynitrite (ONOO^-^) with the help of the endothelial nitric oxide NO synthase (eNOS). The catalase (CAT) and/or glutathione peroxidase (GPX) enzymes maintain the stability of the physiological concentration of hydrogen peroxide. The reaction of excess hydrogen peroxide generates hydroxyl radicals with redox metals can generate hydroxyl radicals. The H_2_O_2_ removal and redox regulation are dominated by peroxiredoxins (PRX). Peroxiredoxins in the figure are represented by green.

## Antioxidant Properties of Exosomes in Various Systems

**Figure 2:**
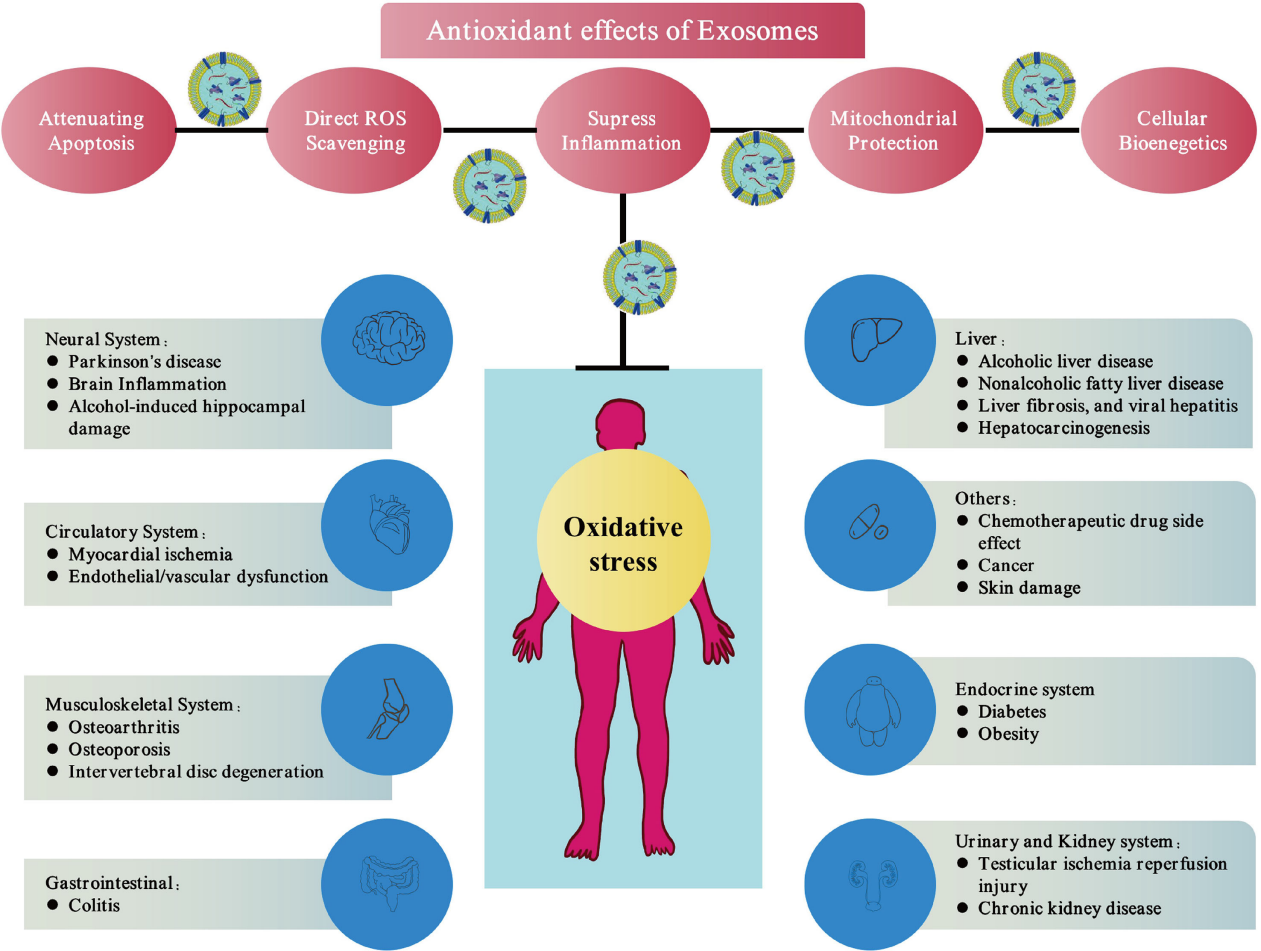
Exosomes therapies *via* antioxidant effects. Exosomes can eliminate free radicals in cells and donate mitochondrial-related proteins, directly or indirectly up-regulate the antioxidant capacity of cells and improve cell bioenergetics. These mechanisms reduce oxidative stress, which show promising antioxidant properties of exosomes.

Exosomes contain various types of lipids, proteins, mRNA and microRNA, etc., which enable the exosomes to as carriers of various signal transduction pathways in cells (67). Exosomes have been found to have therapeutic effects in multiple disorders. In this section, we summarize the anti-oxidative stress activity of exosomes in different systems and the mechanism of their antioxidant function (**Figure 2** and **Table 1**).

**Table 1:** Antioxidant activity of exosomes in disease models.

Application	Model	Exosomes used	Effect of exosome treatment	Antioxidant mechanisms	Reference
Neural system	6-OHDA induced Parkinson’s disease model	Catalase exosomes	Anti-inflammation, significant neuroprotective effects	ROS↓	(26)
Neural system	LPS-induced brain inflammation	Anti-inflammation drugs exosomes	Anti-inflammation effects	ROS↓	(27)
Neural system	Alcohol chronically consuming rats model	MSC-derived exosomes	Reverse alcohol-induced hippocampal oxidative stress	GLT1↑	(44)
Liver system	CCl4-induced liver injury (mice)	Human umbilical cord MSC-derived exosomes	Inhibit oxidative stress-induced apoptosis	ERK1/2 phosphorylation↑ Bcl2↑ SOD↑ ROS↓	(49)
Liver system	CCl4-induced liver injury (mice)	Human umbilical cord MSC-derived exosomes	Reduce oxidative stress, inhibited apoptosis and fibrosis	ROS↓ caspase 3↓	(68)
Liver system	CCl4-induced liver injury and ischemic/reperfusion liver injury (mice)	MSC-derived exosomes	Reduce oxidative stress on the injury-induced liver cells, repair and recover the injured liver tissue	ROS↓	(69)
Liver system	H_2_O_2_ treated human immortalized hepatocytes	HPC-derived exosomes	Prevent oxidative induced cell death of hepatocyte	NRF2↑ GCL↑ ROS↓	(70)
Digestive system	Experimental Colitis (rats)	BMSC-derived exosomes	Attenuate colon Inflammation, oxidative stress and apoptosis	SOD↑ ROS↓ caspase-3, caspase-8 and caspase-9↓	(51)
Cardiovascular system	Unilateral hind-limb ischemia (mice)	Coronary serum exosomes derived from patients with myocardial ischemia	Promote angiogenesis, promoted ischemic injury repair	miR-939-5p↓ VEGF↑ iNOS↓	(50)
Cardiovascular system	Chronic heart failure induced by left coronary artery ligation (rats)	MSC-derived exosomes	Modify myocardial dysfunction	NRF2↑ ROS↓	(71)
Cardiovascular system	Injury model induced endothelial cells	ACE2 induced endothelial progenitor cells exosomes	Protect endothelial cells from injury and apoptosis	ROS↓ NOX2↓	(53)
Cardiovascular system	H_2_O_2_ treated cardiac microvascular endothelial cells (mice)	Hypoxia-pretreated cardiomyocytes exosomes	Reduce the apoptosis and oxidation state of cardiac vascular endothelial cells	CircHIPK3↑ miR-29a induce IGF-1↑	(72)
Cardiovascular system	5/6 NTP induced vascular calcification and ageing mice	VSMC-derived exosomes	Attenuate vascular calcification and ageing	MiR‐204↑ miR‐211↑ BMP2↑	(73)
Musculoskeletal System	Intervertebral disc degeneration (rabbits)	MSC-derived exosomes	Prevent the progression of degenerative changes	Mitochondrial function↑ ROS↓ NLRP3 inflammasome↓	(46)
Musculoskeletal System	Osteoarthritis (mice)	MSC-derived exosomes	Reduce the level of ROS in degenerative chondrocytes, restore mitochondrial dysfunction	Mitochondrial function↑ ROS↓ Inflammation↓	(74)
Musculoskeletal System	Osteoarthritis (mice)	MSC-derived exosomes	Decrease mtDNA damage, increase ATP synthesis, facilitate cartilage regeneration	Mitochondrial function↑ ROS↓ Inflammation↓	(75)
Musculoskeletal System	Chondrocytes obtained from patients diagnosed with advanced OA	Human adipose tissue-derived MSC exosomes	Anti-inflammatory properties in degenerated chondrocytes	iNOS↓	(48)
Musculoskeletal System	Radiation-induced bone loss (mice)	BMSC-derived exosomes	Restore recipient BMSC function, alleviate radiation-induced bone loss	Wnt/β-catenin↑ SOD1↑ SOD2↑ ROS↓	(47)
Endocrine system	Untreated diabetic control wounds	ADSC-derived exosomes	Facilitate faster wound closure, enhance collagen deposition, increase neo-vascularization, decrease oxidative stress	ROS↓	(42)
Endocrine system	Obese mice	Adipocyte exosomes	Attenuate adipose inflammation, decease macrophage number, prevent and treat obesity	αKG↑ STAT3/NF-κB↓	(43)
Skin	H_2_O_2_-stimulated keratinocytes or UV-irradiated mice skin	MSC-derived exosomes	Inhibit oxidative injury, promote antioxidant activity, alleviate oxidative responsiveness	NRF2↑ SOD↑ ROS↓	(76)
Tumor	MCF7-injected tumor (mice)	Camel milk exosomes	Decrease breast tumor progression, induce antioxidant status	SOD↑ ROS↓	(77)
Immune system	CTX induce immuno-toxicity (mice)	Camel milk exosomes	Ameliorate immunosuppression and oxidative stress	SOD↑ ROS↓	(54)
Urinary system	Testicular ischemia-reperfusion injury (rats)	BMSC-derived exosomes	Protect against testicular ischemia-reperfusion injury and apoptosis	SOD↑ ROS↓ caspase 3↓	(78)
Urinary system	Murine hind limb ischemia model	Melatonin-treated MSC-derived exosomes	Improve functional recovery and vessel repair, protect mitochondrial function	miR-4516↑	(79)

6-OHDA, 6-hydroxydopamine; 5/6 NTP, 5/6-nephrectomy plus high phosphate diet treat; ACE2, Angiotensin-converting enzyme 2; ADSC, Adipose-derived stem cell; ATP, Adenosine triphosphate; BMP2, Bone morphogenetic protein 2; BMSC, Bone marrow mesenchymal stem cell; CTX, Cyclophosphamide; ERK1/2, Extracellular-regulated kinase 1/2; GCL, Glutamate cysteine ligase; GLT1, Glutamate transporter 1; HPC, Human hepatic progenitor cell; IGF-1, Insulin-like growth factor 1; iNOS, Inducible nitric-oxide synthase; LPS, Lipopolysaccharide; MSC, Mesenchymal stem cell; mtDNA, Mitochondrial DNA; NF-κB, Nuclear transcription factor kappa B; NLRP3, NOD-like receptor family, pyrin domain-containing 3; NOX2, Nicotinamide adenine dinucleotide phosphate oxidase 2; NRF2, Nuclear factor erythroid 2-related factor 2; ROS, Reactive oxygen species; SOD, Superoxide dismutase; STAT3, Signal transducers and activators of transduction-3; VEGF, Vascular endothelial growth factor; VSMC, Vascular smooth muscle cell; αKG, α-ketoglutarate.

### Nervous System

As life expectancy increases, ageing-related diseases, such as neurodegenerative diseases have further increased. PD is one of the fastest-growing ageing-related neurological diseases in developed countries (80). Brain tissues from the PD patients exhibit reduced levels of oxidoreductase, CAT, SOD, and other antioxidants (81–83). Due to the blood-brain barrier, the commonly used antioxidant catalase cannot be delivered to the brain (84). Application of the emerging nano-delivery systems, have drawbacks like the toxicity of nanomaterials and quick drug removal by the mononuclear phagocyte system (85). Exosomes are thought to have the ability to cross the blood-brain barrier and can avoid clearance by the immune system due to the membrane layer (26, 27).

Two different studies have investigated the application of catalase encapsulated by exosomes in PD. Kojima et al. constructed human MSC-derived exosomes containing catalase mRNA (45). They used 6-hydroxydopamine (6-OHDA) to produce cytotoxic levels of ROS and damage to neurons. The results showed that the exosomes they designed could rescue neurotoxicity and reduce inflammation. The expression of neuro-inflammation-related factors in the brain such as glial fibrillary acidic protein (GFAP) (86), allograft inflammatory factor 1 (Iba1) (87), tumour necrosis factor α (TNFα) (88), and CD11b (26), were attenuated by the exosomes. Haney et al. conducted a similar study. The exosomes derived from mouse macrophages consisting of catalase directly incorporated into the exosomes (26). The exosomal formulations of catalase they produced could be located within the neurons and microglia, confirming that exosomes can cross the blood-brain barrier. At the same time, the exosomal preparation significantly reduces inflammation in the brain and improved the survival of neurons.

In addition to catalase delivery, studies have also reported that anti-inflammatory drugs delivered by exosomes can play a role in oxidative stress regulation (27). Zhuang et al. used exosomes from EL-4 cells (mouse lymphoma cell line) for curcumin encapsulation and used it to treat lipopolysaccharide (LPS)-induced brain inflammation model. The results showed that by intranasal administration, engineered exosomes can significantly inhibit the number of inflammatory microglia.

Apart from the drug encapsulation, exosomes can play an anti-oxidative stress role by themselves in neurodegenerative diseases. Ezquer et al. used ethanol to induce excessive oxidative stress and neuro-inflammation in rats (9). According to previous reports, alcohol can inhibit the levels of glutamate transporter 1 (GLT1), leading to an increase in the number of inflammatory microglia. Intranasal administration of MSC-derived exosomes significantly increased the expression of GLT1 and rescued the brain oxidative stress damage caused by alcohol.

### Digestive System

Increased oxidant stress is recognized as a key factor in most chronic liver diseases, such as viral hepatitis, liver fibrosis, non-alcoholic fatty liver disease, and alcoholic liver disease (89). The progression of hepatocarcinogenesis is often accompanied by the imbalance of intracellular oxidative stress (90). More and more researchers currently use antioxidants as therapeutic agents (91–94). Indeed, there is increasing evidence showing that the therapeutic effects of MSCs are driven by the release of exosomes (16). Moreover, several studies have demonstrated that human MSC may induce tumour growth, whereas MSC-derived exosomes are biologically safe (95).

Several studies have investigated the application of exosomes in liver diseases. Yan et al. isolated human umbilical cord MSC-derived exosomes (hucMSC-Ex) to rescue acute liver injury and liver fibrosis induced by CCl_4_ and H_2_O_2_ (49). They found that hucMSC-Ex relieve CCl4 and H_2_O_2_ induced liver injury both *in vitro* and *in vivo*, which might be related to the delivery of glutathione peroxidase 1 (GPX1) to eliminate excess ROS and inhibition of oxidative stress-induced apoptosis *via* upregulation of extracellular-regulated kinase 1/2 (ERK1/2) and B-cell lymphoma-2 (Bcl-2) and downregulation of the inhibitor kappa B kinase β (IKKβ)/nuclear transcription factor kappa B (NF-κB)/caspase-9/caspase-3 pathway. Knockout of GPX1 in hucMSCs abolished the antioxidant and anti-apoptotic capabilities of HucMSC-Ex and weakened its hepatoprotective effects *in vitro* and *in vivo*. Jiang et al. performed a similar study (68), where a commonly used hepatoprotective agent (bifendate) was compared to exosomes to evaluate the antioxidant effect of exosomes in liver injury. Interestingly, HucMSC-Exs exhibit a stronger antioxidant effect in the pathological process of liver tumours induced by CCl4. Damania et al. showed that MSC-derived exosomes reduced oxidative stress in *in vitro* liver injury models (69).

Hyung et al. confirmed that human hepatic progenitor cell (CdH)-derived exosomes (EXO-hCdHs) significantly reduce the oxidative stress response and delay hepatocyte cell death (70). The data showed that EXO-hCdHs inhibited oxidation-induced cell death in hepatocytes. Consistently, EXO-hCdHs activated nuclear factor erythroid 2-related factor 2 (NRF2) expression and induced downstream regulators. NRF2 is an emerging cellular antioxidant regulator, which can induce the expression of antioxidant-related genes to further regulate cellular oxidative stress (96) and protect against an imbalance of ROS (97).

In addition, exosomes attenuated the severity of colitis (51). The therapeutic effect of BMSC-derived exosomes in colitis is related to the suppression of oxidative disturbance, which is manifested by decreased activities of myeloperoxidase and malondialdehyde (MDA), as well as increased levels of SOD and glutathione. These studies indicate that exosomes are promising candidates for the treatment of oxidative stress-related digestive system diseases.

### Circulatory System

Atherosclerosis, hypertension, and macrovascular disease, blood vessel damage are common in individuals of advanced age (98). Many researchers have demonstrated that excessive oxidative stress can cause endothelial/vascular dysfunction (99). Therefore, reducing oxidative stress is a very important therapeutic strategy for improving vascular function and treating cardiovascular diseases in the elderly.

In the case of hypoxia/reoxygenation (H/R)-induced injury, senescent endothelial cells have a higher rate of apoptosis, excessive ROS, insufficient nitric oxide (NO), upregulated nicotinamide adenine dinucleotide phosphate oxidase 2 (NOX2), downregulate angiotensin-converting enzyme 2 (ACE2) and eNOS, and compromised tube formation ability. The endothelial progenitor cells triggered by ACE2 can protect endothelial cells through the release of exosomes (53). This effect may be due to the presence of miR-18a, which subsequently downregulates the NOX2/ROS pathway.

Ribeiro-Rodrigues et al. found that the exosomes of cardiomyocytes under ischemic conditions can promote the formation of cardiovascular blood vessels (100). In another similar study, Li et al. collected coronary artery serum exosomes from patients with myocardial ischemia and found that this type of exosomes could promote endothelial cell proliferation and migration, and tube formation (50). They also found that exosomes induced by such ischemic conditions had low levels of miR-939-5p. As miR-939-5p has the ability to downregulate the inducible nitric oxide synthase (iNOS) and NO (related to endothelial cell proliferation and tube formation), ischemia-induced exosomes have the ability to promote blood vessel formation.

Exosomes obtained from cardiomyocytes subjected to hypoxia or inflammation have been found to show anti-oxidative stress function. Wang et al. isolated exosomes from the cardiomyocytes preconditioned using hypoxia and it was found that the exosomes obtained after this treatment had a stronger repair capability and significantly upregulated circHIPK3 than in the exosomes derived from unconditioned cells (72). Previous studies have confirmed that the circRNAs can be transferred to target cells *via* exosomes (52). It was also shown that exosomes rich in circHIPK3 can reduce levels of apoptosis and oxidative stress in cardiac vascular endothelial cells (shown by the decrease in MDA levels and increased SOD activity), upregulate the anti-apoptotic protein Bcl-2, and downregulate the pro-apoptotic proteins Bax and cleaved caspase-3.

In another study (71), the authors used TNFα to stimulate cardiomyocytes and isolated the subsequently produced exosomes. The exosomes obtained in this way are rich in a variety of miRNAs (microRNA-27a, microRNA-28a, and microRNA-34a). The results show that such miRNA-rich exosomes can cause an imbalance in the NRF2/antioxidant response element (ARE) signalling pathway. As the NRF2/ARE signalling pathway plays an important role in preventing oxidative damage to the cardiomyocytes (101), the authors believe that this type of exosome is related to the oxidative stress of cardiomyocytes.

In addition to the changes in exosomes caused by hypoxia, ischemia, and inflammation, the body’s hormone levels can also regulate the cardiovascular system through exosomes. Melatonin is a type of indole neuroendocrine hormone necessary for maintaining physiological functions (102). Previous studies have shown that melatonin improves the cardiovascular system through the direct downregulation of excessive ROS and indirect antioxidant activity. A study by Feng et al. found that melatonin could stimulate vascular smooth muscle cells to release exosomes containing miR-204/miR-211, which might be able to target bone morphogenetic protein 2 (BMP2), thereby reducing vascular calcification and ageing (73).

### Musculoskeletal System

Degenerative diseases of the musculoskeletal system, such as IVDD, OA, and osteoporosis, have had a huge impact on society, and the quality of life of middle-aged and elderly people suffering from these diseases is significantly decreased (80).

Previous studies have shown that the onset of IVDD is closely related to ROS and oxidative stress (103). The study by Xia et al. investigated the effect of MSC-derived exosomes on oxidative stress in degenerating intervertebral discs (46). Mitochondria are the primary ROS-producing organelles and are also the source of organelle damage caused by ROS (104). Excessive mitochondrial ROS production can cause cells to lose homeostasis. Exosomes play an important role in mitochondrial communication between cells and can mediate information transmission between cells (75, 105, 106). They found that exosomes could inhibit the H_2_O_2_ induced cell apoptosis and the expression of inflammatory factors (iNOS and interleukin-6). At the same time, exosomes can reduce the production of intracellular ROS and structural abnormalities in mitochondria. This anti-oxidative stress effect may be due to the action of a variety of mitochondria-related proteins in exosomes.

OA is the most common joint disease worldwide (107). Similar to IVDD, many studies have confirmed that inflammation is associated with oxidative stress damage in arthritic chondrocytes which has been reviewed previously (108). Interleukin-1β (IL-1β), a pro-inflammatory factor, stimulates chondrocytes to produce iNOS and NO. Tofiño-Vian et al. found that the use of exosomes derived from adipose tissue-derived MSCs can reduce the levels of nitrite in the medium and the mRNA expression of iNOS in OA chondrocytes (48). Similar to the study of intervertebral discs, Chen et al. investigated the effects of primary chondrocyte exosomes and bone marrow MSC-derived exosomes on the expression of ROS in degenerative chondrocytes in two studies (74, 75). They found that these two types of exosomes can reduce the structural abnormalities of mitochondria and the intracellular ROS production, thereby having a therapeutic effect on cartilage degeneration.

BMSC-derived exosomes have also shown good prospects in bone loss-related diseases. Two studies have found that exosomes can also delay osteoporosis by inhibiting osteoclast metabolism, promote local angiogenesis to prevent femoral head necrosis (109, 110). Zuo et al. found that bone loss caused by radiotherapy is accompanied by excessive oxidative stress, DNA damage, and chromosomal aberrations. BMSC-derived exosomes can reduce the oxidative stress damage of BMSCs caused by irradiation by reducing ROS production, promoting the expression of antioxidant proteins (such as catalase, SOD1, and SOD2) (47), and accelerating DNA repair after radiation.

### Other Systems

In addition to the diseases of the above-mentioned systems, exosomes can also exert anti-cancer effects by regulating abnormal oxidative stress metabolism in the tumour microenvironment. Furthermore, a series of studies have applied exosomes to urinary diseases and immune deficiency diseases caused by oxidative stress.

Badawy et al. found that camel milk-derived exosomes showed anticancer effects (77). The milk-derived exosomes significantly increased the activities of antioxidant enzymes (SOD, GPX, and CAT), and decreased the levels of the lipid peroxidation marker MDA and the expression of the oxidative stress marker iNOS in tumour tissues. Zhuang et al. have also reported that a STAT3 inhibitor delivered by exosomes can efficiently inhibit tumour growth (27).

The oxidative stress-related and immunosuppressive effects of chemotherapeutic drugs are well established (111, 112). In an earlier study, Ibrahim et al. have confirmed that cyclophosphamide (CTX) decreased CAT, SOD, and GPX levels, and induced immunosuppression in rats (54). However, compared with CTX-treated animals, the camel milk exosomes significantly reduced the level of lipid peroxidation marker MDA and increased the activity of antioxidant enzymes SOD, GPX, and CAT. In addition, camel milk exosomes can normalize biochemical and immunological parameters.

The progression in chronic kidney disease often leads to loss of kidney function, resulting in ineffective homeostasis. Yoon et al. reported that exosomes derived from melatonin-treated MSCs (MT exosomes) can significantly improve the pathophysiology of chronic kidney disease (79). They demonstrated that the PrPc protein (a highly conserved and ubiquitous glycoprotein) in exosomes improves the immunomodulatory effect and up-regulates antioxidant proteins in the cell (113, 114), thus improving the regenerative potential of MSCs and attenuating ischemia-induced oxidative stress.

Testicular ischemia-reperfusion injury (IRI) is the main pathophysiological process of surgical reduction after testicular torsion. A large number of oxygen free radicals and inflammatory cytokines play an important role in the pathophysiological process of IRI (115, 116). Zhang et al. found that the treatment of BMSC-derived exosomes can increase the SOD activity of ischemia-reperfusion-injured testicular tissue and decrease MDA content (78). This preliminary study indicated that BMSC-derived exosomes can reduce testicular IRI to protect spermatogenesis.

Diseases such as diabetes and obesity are metabolism-related diseases, which are often accompanied by increased inflammation and oxidative stress (117, 118). For example, in diabetic ulcers, the number of ROS-releasing macrophages and neutrophils increases (119, 120), often leading to wounds that cannot heal. A previous study reported that the amputation rate in diabetic patients was 10-20 times higher than that of non-diabetic patients (121). Shiekh et al. extracted exosomes derived from adipose-derived stem cells (ADSCs) and combined them with a scaffold capable of releasing oxygen (42). The composite structure of the scaffold can significantly enhance the healing of diabetic wounds. At the same time, their research showed that ADSC-derived exosomes can reduce the effects of hyperglycaemia by reducing oxidative stress, thereby increasing the metabolic activity of cells. Exosomes can not only promote the healing of diabetic wounds, but Wang et al. found that exosomes can also reduce skin oxidative stress damage caused by ultraviolet rays (76). In their study, MSC-derived exosomes reduced ROS production, DNA damage, and mitochondrial changes, in a process regulated by the NRF2 defence system.

Low-grade inflammation accompanied by obesity often results in the infiltration of immune cells into adipose tissue (122). The inflammation of adipose tissue leads to the downregulation of α-ketoglutarate (αKG), which is a target for melatonin inhibition of adipose inflammation. A study by Liu et al. demonstrated that the melatonin increased the release of adipose-derived exosomal αKG, which in turn attenuated signal transduction and activation of transduction-3 (STAT3)/NF-κB signalling pathway by its receptor, oxoglutarate receptor 1 (OXGR1), in adipocytes (43). Therefore, melatonin may have the ability to prevent and treat systemic inflammatory diseases caused by obesity through exosomes.

## Role of Exosomes in Inflammation and Degeneration

Under normal physiological conditions classically activated (M1) macrophages secrete pro-inflammatory factors such as TNFα, interleukin-6, and IL-1β, while alternatively activated (M2) macrophages show an anti-inflammatory phenotype (123, 124). Additionally, regulatory T cells (T_regs_) and type 2 T helper (T_h_2) cells secrete anti-inflammatory factors (125). These cells maintain the homeostasis of the immune microenvironment. When cells are stimulated by harmful factors such as toxic chemicals, heat, abnormal pressure, tumours, ageing, or degeneration the cellular inflammatory response triggers a series of reactions (126). The inflammation-related cells secrete exosomes that contain pathogen-related and injury-related molecules and pathogenic antigens (127). In addition, the proteases and glycosidases in these exosomes also cause tissue destruction. This indicates that exosomes play a key role in the process of inflammation (127). Therefore, exosomes are important targets for the treatment of inflammation-related diseases in the future.

In addition to inflammation-related diseases, exosomes also have treatment potential in the degenerative diseases. For example, in PD, glial cell-derived exosomes can transmit beneficial or harmful information to neurons through their internal cargo (128). As exosomes can penetrate the blood-brain barrier, the exosomes released by neuronal cells can be detected in serum or plasma. Since exosomal constituents can provide information about their source cells, exosomes have the potential to become a new type of disease diagnostic markers (129). Moreover, researchers have revealed the relationship between endothelial cell inflammation and brain degeneration by analysing endothelium-derived exosomes isolated from plasma (130).

Many studies have confirmed the ability of MSCs to inhibit inflammation. As MSCs mainly exert biological effects by secreting exosomes, an increasing amount of research is focused on exploring the anti-inflammatory effect of MSC-derived exosomes. The study of this mechanism has shown that MSC-derived exosomes can induce polarization of macrophages from M1 type to M2 type (131–133), thereby changing the phenotype of macrophages from pro-inflammatory to anti-inflammatory. Some investigators have also found that MSC-derived exosomes can regulate the function of T cells, inducing the transition from type 1 T helper (T_h_1) cells to T_h_2 cells (134). MSC-derived exosomes have also become a next-generation treatment strategy for degenerative diseases such as PD (135), OA (136), IVDD (137), and macular and retinal degeneration (138). These abilities of MSC-derived exosomes are mainly exerted by the constituent proteins, mRNA, and miRNA, which attenuate chronic inflammation, reduce apoptosis and stimulate proliferation (139).

## Future Perspectives

According to the presented literature review, it can be seen that the anti-oxidative stress properties of exosomes have been confirmed in multiple systems, which proves the potential of exosomes as a therapeutic agent against oxidative stress. However, before further application, the following hurdles still need to be overcome. First, exosomes are highly heterogeneous, showing a high degree of diversity in the size of exosomes derived from different cell sources, the internal biological components, and their effect on the function of the recipient cells (28). Exosomes derived from the MSCs, a common source of exosomes, exhibit huge differences in the expression of MSC membrane markers and proteomic characteristics even when derived from the same tissue (140). The cell microenvironment and internal biology may also affect the content of exosomes and their biomarkers (29–31, 52). Based on an in-depth understanding of their heterogeneity, it is necessary to accurately characterize the exosomes used in different experiments.

Secondly, how exosomes affect the function of recipient cells requires further study. At present, most studies have focused on the components of exosomes (such as proteins, miRNAs, and circRNAs) that exert antioxidant effects on recipient cells. However, the behaviour of these components after entering the cell needs further investigation. The location of these components in the cell and whether they are degraded by lysosomes is unknown. Existing studies have shown that exosomes may be taken up by cells in different ways. For example, human melanoma cells take up cargo in exosomes through membrane fusion (21), while in neurosecretory PC12 cells (derived from rat adrenal medullary tumours), exosomes are more dependent on clathrin-dependent endocytosis for uptake (19). How different types of exosomes enter the cell and deliver cargos (i.e., membrane fusion, macropinocytosis, phagocytosis, and clathrin-dependent endocytosis), and what factors affect this process, need to be further investigated (141, 142).

Thirdly, although it is assumed that exosomes can functionally deliver their internal miRNA to target cells there is still a lack of direct evidence for exosomes mediated miRNA transfer. As lipoproteins are inevitably mixed with exosomes in the current exosome extraction process and lipoproteins also have the function of transferring miRNA (143), it is necessary to purify exosomes to determine their efficiency of miRNA delivery.

Lastly, the current research on exosomes is limited to cellular or animal models, and there are very few human studies. Although the studies reviewed in this article demonstrate the potential of exosomes to resist oxidative stress at the cellular and organ levels, more clinical trials need to be conducted to test the feasibility of this strategy.

## Conclusion

The findings summarized here demonstrate that MSC-derived exosomes and exosomal formulations show excellent antioxidant properties. Growing evidence shows that exosomes can eliminate excessive ROS in cells and deliver mitochondrial protective proteins, thereby improving the antioxidant capacity of cells and enhancing cell viability. Future research should focus on elucidating the specific differences in the antioxidant mechanisms of exosomes in different diseases. Similarly, the in the future, research should be focused on targeted tissue engineering of exosomes, such as increasing the content of specific antioxidant enzymes or mitochondria related proteins in exosomes to further enhance the efficacy of exosomes. The MSC-derived exosome-based therapy has promising application prospects in multiple systemic diseases.

## Author Contributions

Conceptualization, PC and CX. Writing—review and editing, CX, ZD, YJ, and PC. All authors contributed to the article and approved the submitted version.

## Funding

The study was supported by the National Key R&D Program of China (Grant No. 2020YFC1107104), the National Nature Science Fund of China (Grant No. 81802147, and 81772387), and the Natural Science Fund of Zhejiang Province (Grant No. LY21H060002).

## Conflict of Interest

The authors declare that the research was conducted in the absence of any commercial or financial relationships that could be construed as a potential conflict of interest.

## Publisher’s Note

All claims expressed in this article are solely those of the authors and do not necessarily represent those of their affiliated organizations, or those of the publisher, the editors and the reviewers. Any product that may be evaluated in this article, or claim that may be made by its manufacturer, is not guaranteed or endorsed by the publisher.
